# Primary Melanoma Histopathologic Predictors of Sentinel Lymph Node Positivity: A Proposed Scoring System for Risk Assessment and Patient Selection in a Clinical Setting

**DOI:** 10.3390/medicina59111921

**Published:** 2023-10-30

**Authors:** Jelena Jeremić, Kristina Radenović, Milana Jurišić, Branko Suđecki, Milana Marinković, Jovan Mihaljević, Ivan Radosavljević, Milan Jovanović, Marina Stojanović, Nataša Milić, Vedrana Pavlović, Milan Stojičić, Zorka Inić, Marko Jović

**Affiliations:** 1 Clinic for Burns, Plastic and Reconstructive Surgery, University Clinical Center of Serbia, 11000 Belgrade, Serbiadrivanradosavljevic@gmail.com (I.R.);; 2 Faculty of Medicine, University of Belgrade, 11000 Belgrade, Serbiazorkainic@gmail.com (Z.I.); 3 Center for Anesthesia, University Clinical Center of Serbia, 11000 Belgrade, Serbia; 4 Institute for Medical Statistics and Informatics, 11000 Belgrade, Serbia; 5 Surgical Oncology Clinic, Institute for Oncology and Radiology of Serbia, 11000 Belgrade, Serbia

**Keywords:** melanoma, sentinel lymph node biopsy, SLNB, Breslow, mitosis, ulceration, scoring system

## Abstract

*Background and Objectives:* The careful selection of adequate SLNB candidates not only aims at reducing the surgical risk while identifying SLN metastasis, but also plays a crucial role in identifying the patients eligible for adjuvant therapy. Objectives: The purpose of our study was to investigate the clinical and histologic aspects of primary melanomas that correlate with the likelihood of a positive SLNB result. *Materials and Methods*: A total of 101 primary melanoma patients who underwent sentinel lymph node biopsies were included in the study. General patient demographics were obtained as well as localization and melanoma-specific characteristics of primary melanoma from histologic reports in addition to data derived from SLNB melanoma histopathology reports. *Results*: The patients with positive SLN results had a statistically significant increased Breslow thickness (3.8 mm vs. 1.97 mm, *p* = 0.002), higher mitotic index rate (5/mm^2^ vs. 2/mm^2^, *p* = 0.009), as well as the presence of ulceration (68.4% vs. 31.6%, *p* = 0.007). Univariate regression analysis showed the Breslow thickness (*p* = 0.008), the mitotic index rate (*p* = 0.054), the presence of ulceration (*p* = 0.009), as well as the pT3-4 stage (*p* = 0.009) to be significant predictors of SLN positivity. The optimal cut-off values for Breslow thickness and the number of mitoses scores were determined based on ROC curve analysis. Using the Breslow thickness, mitotic index rate, presence of ulceration, and pT3-4 stage significant coefficients from the univariate regression model, a chance prediction score was developed. *Conclusions:* The newly developed and proposed scoring system can aid in patient selection for SLN biopsy by facilitating a more efficient risk assessment in the detection of lymph node metastases in melanoma patients.

## 1. Introduction

Even though melanoma accounts for 1.7% of all cancer diagnoses worldwide, it is considered to be one of the most fatal skin carcinomas, with the highest death rate among the skin tumors [1,2,3]. With an estimated 106.110 new diagnoses and 7.180 deaths in 2021, a dramatic increase in incidence has been recorded in recent decades, especially in regions with fair-skinned populations [1,2,4,5]. Since 1970, the survival rates have improved greatly, with a current 10-year survival rate of 90% [6]. However, mortality from advanced disease forms still remains significant: patients with regional lymph node involvement are found to have a 5-year survival rate between 40% and 78% [6]. Histopathological diagnoses with a higher Breslow index and the presence of ulcerations are both found to indicate a more aggressive form of the disease with a higher risk of local and distant metastases and a generally worse prognosis [7]. The survival rate of melanoma is highly correlated with the stage of the disease at the time of diagnosis, which is, among others, determined by the presence of metastases in the lymph nodes.

The potential for the presence of metastases in the local nodal basin or elsewhere can be predicted via the biopsy and histology of the sentinel lymph node (SLN), the first lymph node involved in the lymphatic spread of melanoma. Knowing the status of regional lymph nodes carries extremely valuable prognostic information that is helpful in setting a strategy for regional disease control and in the selection of patients who would benefit from adjuvant therapy [8]. The identification rate for SLNB with radiocolloid in melanoma patients was found to be as high as 99% in the literature, while other authors reported false negative results in 12.5% of cases [9]. Possible complications, such as seroma, hematoma, surgical site infection, neural damage, or lymphoedema, might arise following SLNB; hence, the procedure is not risk-free, and the careful and appropriate selection of patients is critical [9].

According to the American Joint Committee on Cancer (AJCC), eight edition, and National Comprehensive Cancer Network (NCCN) recommendations, the indications for SLNB are having intermediate-thickness melanomas, being at stages T2 and T3 (1 mm to 4 mm) with thinner melanomas (0.8–1 mm or <0.8 mm with ulceration), as well as thick melanomas (>4 mm); these should include an open discussion with the patient about potential risks and benefits of the procedure [7,10,11]. The role of SLNB in thick melanomas (Breslow thickness >4 mm) is debatable due to the possible risk of distant metastases regardless of the lymph node status ascribed to possible bloodborne dissemination [11]. On the other hand, the chance of having positive SLN in thin melanomas in the literature runs at about 5% and is still an important subject of research [9,12].

Several clinical and histologic features have been shown to be predictive of SLN positivity. Some studies have shown that the incidence of positive SLN after SLNB mainly depends on the thickness of the primary tumor and ranges from 15 to 20% in the literature. The incidence of positive SLN after SLNB in T4 tumors is considered to be 35–40%, while for T1 lesions, the incidence is 5–7.8%, as descriptively presented [13,14,15]. In addition to the thickness of the tumor according to Breslow, the presence of ulceration, the level of the mitotic index, being young, lymphovascular invasion, and tumor localization (trunk) are all found to be prognostic factors associated with an increased risk of SLN positivity [11,16,17,18,19]. Numerous studies have been conducted researching characteristics that may accurately identify the patients at risk of developing melanoma SLN metastasis as well as to select those who are appropriate candidates in terms of surgical complication risks, aiming at determining the best risk–benefit patient population [16].

Additionally, the careful selection of adequate SLNB candidates not only aims at reducing the surgical risk while identifying SLN metastasis, but also plays a crucial role in identifying the patients eligible for adjuvant therapy. The primary analysis after a median follow-up of 34 months by the German Dermatology Cooperative Oncology Group (DeCOG-SLT) did not show a benefit in terms of distant metastasis-free survival (DMFS), overall survival (OS), and recurrence-free survival (RFS) for patients undergoing CLND immediately post-SLNB, while the final analysis of the DeCOG-SLT study performed 3 years after the inclusion of the last patient also showed similar results [20]. Additionally, after a median follow-up of 43 months, the MSLT-II study did not identify any survival advantage for the SLNB-positive patients who received CLND [21]. These results indicate SLNB as an important diagnostic procedure in terms of staging and adjuvant therapy, rather than its therapeutical benefit. Nonetheless, many ongoing studies aim to further refine the criteria for SLNB.

The purpose of our study was to investigate the clinical and histologic aspects of primary melanomas that correlate with the likelihood of having a positive SLN result to develop and propose an easy-to-use scoring system that predicts SLN positivity based on the presence of multiple independent predictors, which is used for the more efficient selection of patients in a clinical setting.

## 2. Materials and Methods

This retrospective study was conducted at the Clinic for Burns, Plastic and Reconstructive Surgery, University Clinical Center of Serbia, Belgrade. After the approval of the Institutional Review Board (number 602/1, 30 December 2021), data were extracted from patients’ medical records and the histopathology results. All samples were obtained from the histopathological reports from the Institute of Pathology, Faculty of Medicine, University of Belgrade, and analyzed by a pathologist with experience in the analysis of melanocytic lesions.

### 2.1. Patient Selection, Inclusion and Exclusion Criteria

This study included primary melanoma patients treated between 1 January 2017 and 31 March 2022. All the patients were treated according to the current recommendations of the American Joint Committee on Cancer (AJCC) 2017, 8th edition, as well as per the National Comprehensive Cancer Network (NCCN) guidelines. The inclusion criteria were all melanoma patients without clinically or radiologically verified alteration of lymph nodes and/or distant metastases. Patients with a tumor thickness of more than 0.8 mm were included, including patients with microsatelitosis in the histopathological reports. Patients treated for primary melanomas of less than 0.8 mm thickness were also included if ulceration or other risk factors were present. Patients treated for melanoma with satellite, in-transit, or distant metastases, as well as previously treated patients for melanoma or other non-skin related malignancies, patients on immunosuppressive therapy, patients who have already undergone extensive targeted lymph basin surgery, and patients who have undergone primary wide excision were excluded from the study.

### 2.2. Extracted Patient Data

General patient demographics were obtained, such as age and sex, as well as date of biopsy, localization, and melanoma-specific characteristics of primary melanoma, e.g., subtype, Breslow thickness, Clark grade, mitotic index rate, and ulceration status. According to the primary distribution, all the melanomas were divided into 4 localizations: (a) head and neck, (b) trunk, (c) upper extremities, and (d) lower extremities. All the samples were histologically divided into 4 large groups: (a) superficial spreading melanoma (SSM), (b) nodular (NOD), (c) lentigo maligna (LMM), and (d) other (acral lentiginous (ALM), nevoid, spitzoid, dermal, desmoplastic, meltump, malignant blue nevus, polypoid, and Reed nevus-like melanoma). The Breslow thickness was divided into four categories, according to the guidelines of the 8th edition of the AJCC from 2017: <1 mm, 1.01–2 mm, 2.01–4 mm, and >4 mm. The Clark depth was categorized into five levels (1–5). The mitotic index rate was calculated using the hot spot/mm^2^ method, while ulceration status was marked as present versus absent.

Sentinel lymph nodes were mapped via lymphoscintigraphy using technetium 99^m^Tc-sulfur radiocolloid. Data derived from SLNB melanoma histopathology reports included the size of the largest metastatic deposit, the number of positive sentinel nodes, and the localization of metastasis in the sample. The localization of the metastatic deposit in the sentinel lymph node was categorized as parenchymatous, capsular, subcapsular, extensive, or parenchymal-subcapsular. The sentinel lymph node tumor burden was classified according to the Rotterdam criteria related to the diameter of the largest metastatic deposit (<0.1 mm; 0.1–1 mm; >1 mm). The clinical stage of the disease was determined based on the thickness of the tumor according to Breslow (T), the status of the lymph nodes (N), and the presence or absence of distant metastases (M).

### 2.3. Statistical Analysis

Descriptive statistics, including means, medians, standard deviations and percentiles for numerical variables, and numbers and percentages for categorical variables, were used to characterize the study sample. No imputation methods were used in the analysis. Associations between the categorical data were evaluated using Pearson’s chi-square test. Student’s *t*-test or the Mann–Whitney U test was used to evaluate the differences between the positive and negative sentinel biopsy numerical data. Univariate logistic regression analysis was used to determine factors related the prediction rate of positive sentinel biopsy. The results are expressed as relative risk and corresponding 95% confidence intervals (CI). Model discrimination performance was tested using sensitivity, specificity, positive, and negative predictive values. The C statistic, representing the area under the receiver operating characteristic curve (ROC), was used for the overall assessment of the predictive model. In all analyses, the level of statistical significance was set at *p* ≤ 0.05. For the statistical analysis, the SPSS version 25 statistical software (Chicago, IL, USA) was used.

## 3. Results

A total of 101 patients underwent a sentinel lymph node biopsy. A total of 59.4% were male, while 40.6% were female. The mean patient age was 56.26 ± 15.09 years (the youngest patient was 16 years old, and the oldest was 86). All the patients were divided into three age groups: younger than 40, 41–60, and older than 60, with the majority of patients being in the older-than-60 group (16.8%, 37.6%, and 45.5%, respectively). The characteristics of the patients included in the study, as well as the histopathological characteristics of primary melanoma, are presented in Table 1. The clinical and histological characteristics of the sentinel lymph nodes are shown in Table 2.

Patients with positive SLN results had a statistically significant increased Breslow thickness (3.8 mm vs. 1.97 mm, *p* = 0.002), higher mitotic index rate (5/mm^2^ vs. 2/mm^2^, *p* = 0.009), and a higher percentage of ulcerated lesions (68.4% vs. 31.6%, *p* = 0.007) than the patients with negative SLN results did, aligning with a higher proportion of pT3-T4 cases among the patients with positive SLN results (48.0% vs. 84.2%; *p* = 0.005). Conversely, sex, age, the anatomic site of primary melanoma, and Clark levels were not significantly associated with SLN positivity (*p* > 0.05). Nodular melanoma was the most prevalent histopathological subtype in the SLN-positive group (47.4% vs. 26.6%), although this finding did not reach statistical significance (*p* = 0.78). The comparison between the SLN-positive and SLN-negative groups is shown in Table 3.

**Figure 1:**
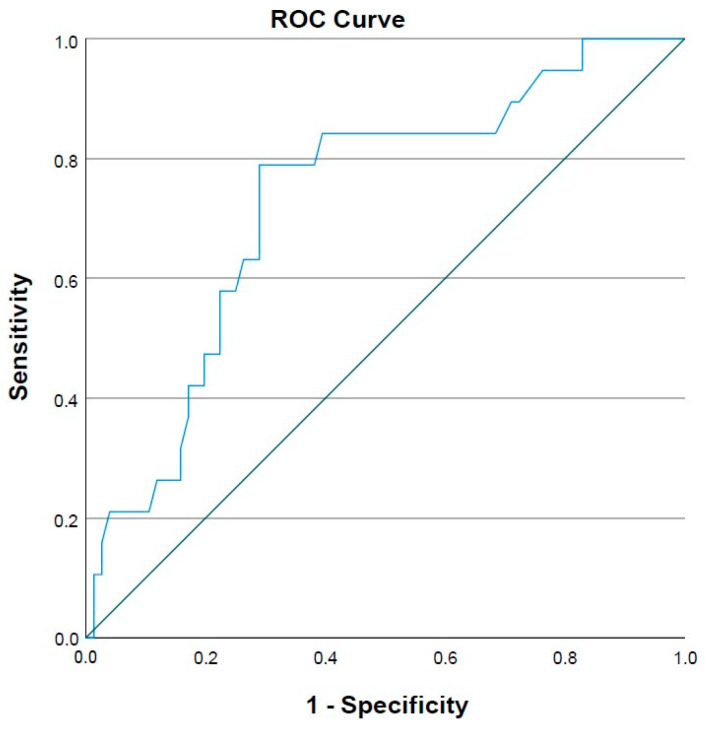
The ROC curve for the Breslow thickness in sentinel lymph node positivity showing the optimal cut-off values for Breslow thickness > 2 mm; ROC curve—receiver operating characteristic curve.

**Figure 2:**
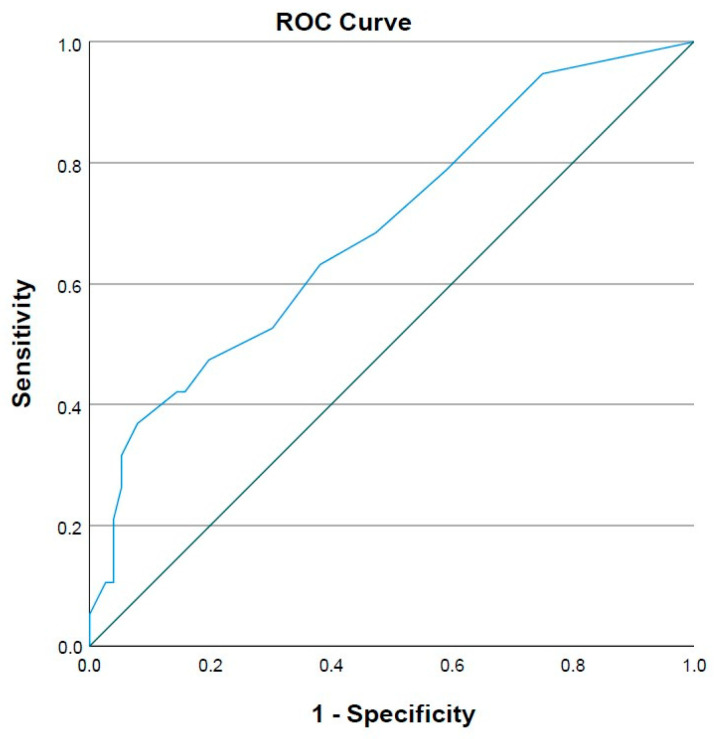
The ROC curve for the number of mitoses in sentinel lymph node positivity showing the optimal cut-off values for mitotic index rate score > 3; ROC curve—receiver operating characteristic curve.

Figure 1 shows the ROC curve for Breslow thickness in SLN positivity with an area under the curve (AUC) Breslow value of 0.731 (*p* = 0.002). Figure 2 shows the ROC curve for the mitotic rate index in SLN positivity with an AUC for the number of mitoses of 0.691 (*p* = 0.01). The optimal cut-off values for Breslow thickness > 2 and mitotic index rate scores > 3 were determined based on the ROC curve analysis.

The diagnostic performance of the Breslow thickness, mitotic index, presence of ulcerations, as well as pT3-4 stage in predicting SLN positivity was tested, and the sensitivity, positive predictive value (PPV), specificity, and negative predictive value (NPV) are shown in Table 4.

The univariate regression analysis showed the Breslow thickness (*p* = 0.008), mitotic index rate (*p* = 0.054), presence of ulcerations (*p* = 0.009), and pT3-4 stage (*p* = 0.009) to be significant predictors of SLN positivity. In a multivariate regression model, the most significant independent predictor was the pT3-4 stage (*p* = 0.009) (Table 5).

### The Scoring System

**Figure 3:**
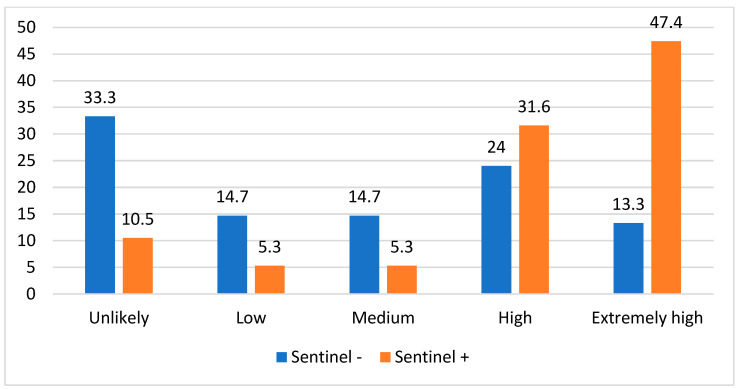
The distribution of the SLN positivity scores in our study population.

Using the significant Breslow thickness, mitotic index rate, presence of ulceration, and pT3-4 stage coefficients from the univariate regression model, the chance prediction score was developed. The newly developed SLN positivity score was determined by assigning the chance of SLN positivity according to the presence of the predictors. A score of 0 presents an unlikely chance of SLN positivity, while there is a low chance (score 1) if one predictor is present, a medium chance (score 2) if two predictors are identified, a high chance (score 3) if there are three predictors, and an extremely high chance (score 4) with all four predictors present. In our research, we found that patients exhibited varying rates of positive SLNB results based on their scores: 10.5% of those with a score of 0, 5.3% for patients with scores of 1 and 2, 31.6% for patients with scores of 3, and 47.4% for those with a score of 4 (*p* = 0.008). The distribution of the SLN positivity scores in our study population is shown in Figure 3.

## 4. Discussion

Great efforts have been put into the refinement of criteria for the careful identification of patients benefiting from SLNB. While several guidelines have been accepted worldwide, various factors still remain inconclusive, such as thin melanomas (<0.8 mm), thick melanomas (>4 mm), SLNB in senior patients, as well as some histologic subtypes of melanoma. All the protocols agree on offering SLNB to patients with more than a 5% chance of having a positive SLNB result [11]. Many studies aimed at further identifying the predictors of SLNB positivity and personalizing the treatment approach for melanoma patients [9]. With many protocols available to clinicians, further refinement in terms of accessibility and efficacy could be a valuable step forward, such as scoring the predictors based on the known chances.

While the AJCC, seventh edition, included the mitotic rate index as an important predictor of SLNB positivity, the AJCC, eighth edition, did not find the mitotic rate index as a valuable predictor when presented as a dichotomous variable (<1 mitosis/mm^2^ vs. ≥1 mitosis/mm^2^) [22]. Still, many authors found the mitotic rate index to be an important predictor when set at different thresholds, which is in accordance with our findings. In our study, the patients with positive SLN results had a higher mitotic rate index (5 vs. 2; *p* = 0.009), with the mitotic rate index being a significant predictor of SLNB positivity (*p* = 0.05). Santos et al. reported a statistically significant association between the mitotic rate index and SLN positivity in a group of people with melanomas ranging from 1 to 4 mm in thickness (*p* = 0.034), while Rodriguez et al. identified hazard ratios statistically significant for the association of a mitotic rate index >3 mm^2^ and SLNB positivity [23,24]. Additionally, after pooling the data, the most recent meta-analysis found a mitotic rate index greater than 0/mm^2^ to be a statistically significant predictor of positive SLNB (adjusted OR 1.63 (95%CI 1.13–2.36)) [9]. While the mitotic rate index remains an independent risk factor in the AJCC, eighth edition, the NCCN recommends discussing SLNB with the patients with a mitotic rate of >2/mm^2^, as it presents an important predictor of SLNB positivity per their recommendations as well as per our scoring system [25].

The presence of ulcerations has been widely accepted as a predictor of SLNB positivity found in all guidelines and by most authors in independent, single-center studies, as well as in our study [9,22,24]. Furthermore, the presence of ulcerations in thin melanomas was found to be the strongest predictor of positive SLN results when the data were pooled [9]. Additionally, when assessing SLNB in thick melanomas, Ribero et al. found the non-SLNB receiving group (the observation group) had the same prognosis as the positive-sentinel lymph node group, while the SLNB receiving group had more favorable outcomes than the SLNB-positive and the observation groups did [26]. White et al. found the absence of ulceration and a lower mitotic index to be favorable prognostic signs in thick melanomas, with the better overall survival rate of these patients. While most recommendations are based on the possibility of bloodborne dissemination or guaranteed lymphatic invasion in thick melanomas, factors such as the presence of ulceration or a high mitotic index are proposed as more important risk factors than the tumor thickness itself is when making decisions regarding SLNB [27]. Nevertheless, the presence of ulcerations is an important indicator of the possible necessity of SLNB in various inconclusive, unofficially defined scenarios as per current guidelines, such as in thin (Breslow > 0.8 mm) as well as thick (Breslow < 4 mm) melanomas, which is similar to our study.

A Breslow thickness >2 mm was found to be a significant predictor of positive SLN results in our study. While the AJCC, eighth edition, and the NCCN recommendations based on a fairly large population sample advise SLNB to patients with a Breslow thickness >0.8 mm with ulceration and a Breslow >1 mm with or without ulceration, other authors report different thresholds for Breslow thickness as an independent risk factor [28]. Nevertheless, Breslow thickness remains a cornerstone of melanoma staging and metastasis prediction. The results obtained in our study are most likely influenced by the size or distribution of the population sample. For the proposition of the newly developed scoring system, one point was assigned to the patients with a Breslow thickness > 2 mm, with additional studies necessary for reevaluation of the Breslow threshold and further refinement of the scoring system.

The inclusion of pT staging enhances the scoring system by consolidating histopathological features, such as Breslow thickness and ulceration status, into a single variable. This facilitates a more structured categorization and assists in identifying patients who would derive the greatest benefit from SLNB.

The choice of patients eligible for SLNB should be based on the existing globally accepted protocols, such as the AJCC or the NCCN, which include the most common, evidence-based features of melanoma patients. Our scoring system is proposed for the quicker and more efficient evaluation of patients in unconventional circumstances, such as patients with desmoplastic, acral melanoma, senior population, or in facilities with limited resource settings (developing countries, etc.). Certain histologic subtypes show a variable predisposition for SLN metastasis. In pure desmoplastic melanomas, SLNB positivity was found in 5.4% of cases, while in mixed desmoplastic melanomas, the rate of SLNB positivity was 13.8% [29]. Conversely, acral lentiginous melanoma was found by some authors to be an independent risk factor for SLN positivity [22,30]. Based on the SLN positivity rates of acral and non-acral melanomas, Kato et al. reported that the association between tumor thickness and the likelihood of SLN positivity may not be linked to the histologic subtype [31]. Age has been discussed by many authors, with the study results implying that being young increases both the SLNB positivity rates as well as the survival rates, whereas elderly patients are less likely to be SLNB-positive, but have a worse prognosis [32,33]. Other single-center studies report higher SLNB positivity rates in senior patients as well as higher SLNB surgical complication rates, while some authors express the benefits of SLNB in senior population with regard to staging and exploring the possibilities of adjuvant therapy [3,34]. Thus, certain clinical scenarios could still be considered a grey area in SLNB decision making. Additionally, even though SLNB is a relatively safe procedure when performed by an experienced surgeon, surgical complications, such as lymphedema, seroma, a hemorrhage, and infection, are some of the described complications. Upcoming pursuits to personalize treatments as well as make more efficiently decisions regarding melanoma diagnostics, staging, and therapy are necessary for identifying patients to whom such a procedure provides the most benefit with the least risk [35].

The score developed based on our results aims at simplifying decision making in a clinical setting, though it is in need of further refinement.

### Study Limitations

This study is mainly limited by its retrospective design. Additionally, the study population may be susceptible to a selection bias, as it relied mostly on patient referrals to a tertiary institution. The selection of patients for SLNB was conducted at a single center, based on AJCC, eighth edition, criteria and further clinical judgement, limiting the population sample of thin as well as thick melanomas and omitting the further stratification of melanoma characteristics. The above-mentioned reasons could have attributed to our discovery of the 2 mm threshold for Breslow thickness as a predictor of SLN positivity. The small sample size resulted in only 14 patients with melanomas < 1 mm, rendering further statistical analysis using a 1 mm Breslow cut-off unfeasible. This limitation may also partly be due to the significant proportion of our cohort including patients treated over the past three years, including the period during and after the COVID-19 pandemic, which could have influenced the data distribution of our sample. One of the main limitations of this study is the results for score 0, which still identifies 10.5% of patients with a positive SLNB, meaning there are still patients invisible to this scoring system. As previously stated, these results are likely influenced by the small population sample as well as the distribution of the population in the sample. Further validation of the proposed score is necessary on a larger population in a multicentric design, as is the refinement of each individual component of the score and its thresholds.

## 5. Conclusions

This newly developed and proposed scoring system can aid in patient selection for SLN biopsies by facilitating the more efficient risk assessment for the detection of lymph node metastases in melanoma patients. Further research is needed to refine the thresholds of SLN positivity predictors as well as validate this scoring system’s effectiveness.

## Figures and Tables

**Table 1:** Demographic and histopathological characteristics of primary melanoma.

Variables	*n* = 101 (100%)
Age (mean ± SD)	56.26 ±15.1
Age groups:	
<40	17 (16.8%)
40–60	38 (37.6%)
>60	46 (45.5%)
Sex:	
Male	60 (59.4%)
Female	41 (40.6%)
Localization of primary melanoma:	
Head and neck	16 (16.2%)
Torso	40 (40.4%)
Upper extremities	21 (21.2%)
Lower extremities	20 (20.2%)
Acral	2 (2.0%)
Melanoma subtype:	
Superficial spreading	58 (59.2%)
Nodular	30 (30.6%)
Lentigo maligna	3 (3.1%)
Acral	2 (2.0%)
Other	5 (5.1%)
Breslow thickness in mm (median, 25th–75th percentile)	2.20 (1.23–4.50)
Breslow thickness:	
<1 mm	14 (14.7%)
1.01–2 mm	29 (30.5%)
2.01–4 mm	26 (27.4%)
>4 mm	26 (27.4%)
Clark level:	
II	5 (5.3%)
III	34 (35.8%)
IV	50 (52.6%)
V	6 (6.3%)
pT staging:	
T1–T2	42 (44.7%)
T3–T4	52 (55.3%)
Mitotic index rate (median, 25th–75th percentile):	3.00 (1.00–6.00)
Ulceration present:	
Yes	39 (41.1%)
No	56 (58.9%)

**Table 2:** Clinical and histological characteristics of sentinel lymph nodes. SLN—sentinel lymph node.

Variables	*n* = 101 (100%)
Nodal metastasis present:	
Yes	19 (18.8%)
No	82 (81.2%)
SLN localization:	
Axilla	54 (53.5%)
Groin	26 (25.7%)
Head & Neck	17 (16.8%)
Interval Nodes	4 (4.0%)
Diameter of SLN metastasis in mm (median, 25th–75th percentile)	1.20 (0.80–2.60)
Microanatomic SLN metastasis localization:	
Subcapsular	3 (16.7%)
Parenchymal	8 (44.4%)
Both structures	7 (38.9%)
Extracapsular spreading of metastatic deposits present	
Yes	2 (11.1%)
No	16 (88.9%)

**Table 3:** The comparison between SLN positive and negative groups.

Variables	SLN Positive	SLN Negative	*p* Value
	*n* = 19 (18.8%)	*n* = 82 (81.2%)	
Age (mean ± SD)	56.21 ± 13.48	56.27 ± 15.52	
Age groups:			0.548
<40	2 (10.5%)	15 (18.3%)	
41–60	9 (47.4%)	29 (35.4%)	
>60	8 (42.1%)	38 (46.3%)	
Sex:			0.160
Male	14 (73.7%)	46 (56.1%)	
Female	5 (26.3%)	36 (43.9%)	
Primary melanoma localization:			0.912
Head and Neck	3 (15.8%)	13 (16.3%)	
Torso	9 (47.4%)	31 (38.8%)	
Upper extremities	4 (21.1%)	17 (21.3%)	
Lower extremities	3 (15.8%)	17 (21.3%)	
Acral	0 (0.0%)	2 (2.5%)	
Melanoma subtype:			0.208
Superficial Spreading	8 (42.1%)	50 (63.3%)	
Nodular	9 (47.4%)	21 (26.6%)	
Lentigo maligna	0 (0.0%)	3 (3.8%)	
Acral	0 (0.0%)	2 (2.5%)	
Others	2 (10.5%)	3 (3.8%)	
Breslow thickness (median, 25th–75th percentile)	3.80 (2.90–7.00)	1.97 (1.20–3.27)	0.002
Breslow thickness:			0.032
<1 mm	1 (5.3%)	13 (17.1%)	
1.01–2 mm	2 (10.5%)	27 (35.5%)	
2.01–4 mm	7 (36.8%)	19 (25.0%)	
>4 mm	9 (47.4%)	17 (22.4%)	
Clark level:			0.390
II	0 (0.0%)	5 (6.6%)	
III	5 (26.3%)	29 (38.2%)	
IV	13 (68.4%)	37 (48.7%)	
V	1 (5.3%)	5 (6.6%)	
pT staging:			0.005
T1–T2	3 (15.8%)	39 (52.0%)	
T3–T4	16 (84.2%)	36 (48.0%)	
Mitotic index rate (median, 25th–75th percentile)	5.00 (2.00–12.00)	2.00 (0.25–5.00)	0.009
Ulceration present:			0.007
Yes	13 (68.4%)	26 (34.2%)	
No	6 (31.6%)	50 (65.8%)	

**Table 4:** Diagnostic performance of prediction tests. (Sn—sensitivity; PPV—positive predictive value; Sp—specificity; NPV—negative predictive value).

Predictors for SLN Positivity	Sn	PPV	Sp	NPV
Breslow thickness	84.2	30.8	52.6	93.0
Mitotic index	63.2	29.3	61.8	87.0
Presence of ulceration	68.4	33.3	65.8	89.3
pT3-4	84.2	30.8	50.2	92.9
Nodular subtype	47.4	30.3	73.4	85.3

**Table 5:** Univariate logistic regression analysis with SLN positivity as dependent variable.

Variable	OR	95% CI for OR	*p*
Univariate analysis			
Nodular subtype	2.486	0.888–6.960	0.078
Breslow thickness	5.926	1.595–22.023	0.008
Mitotic index rate	2.778	0.981–7.866	0.054
Ulceration present	4.167	1.419–12.235	0.009
pT3-4 stage	5.778	1.553–21.493	0.009
Multivariate analysis			
pT3-4 stage	5.778	1.553–21.493	0.009

## Data Availability

Not applicable.

## References

[1] Matthews N.H., Li W.-Q., Qureshi A.A., Weinstock M.A., Cho E., Ward W.H., Farma J.M. (2017). Epidemiology of Melanoma. Cutaneous Melanoma: Etiology and Therapy.

[2] American Cancer Society Key Statistics for Melanoma. https://www.cancer.org/cancer/types/melanoma-skin-cancer/about/key-statistics.html.

[3] Bobircă F., Tebeică T., Pumnea A., Dumitrescu D., Alexandru C., Banciu L., Popa I.L., Bobircă A., Leventer M., Pătrașcu T. (2023). The Characteristics of Sentinel Lymph Node Biopsy in Cutaneous Melanoma and the Particularities for Elderly Patients—Experience of a Single Clinic. Diagnostics.

[4] Aitken J.F., Elwood M., Baade P.D., Youl P., English D. (2010). Clinical Whole-Body Skin Examination Reduces the Incidence of Thick Melanomas. Int. J. Cancer.

[5] Ferlay J., Soerjomataram I., Dikshit R., Eser S., Mathers C., Rebelo M., Parkin D.M., Forman D., Bray F. (2015). Cancer Incidence and Mortality Worldwide: Sources, Methods and Major Patterns in GLOBOCAN 2012. Int. J. Cancer.

[6] Balch C.M., Gershenwald J.E., Soong S.-J., Thompson J.F., Atkins M.B., Byrd D.R., Buzaid A.C., Cochran A.J., Coit D.G., Ding S. (2009). Final Version of 2009 AJCC Melanoma Staging and Classification. J. Clin. Oncol. Off. J. Am. Soc. Clin. Oncol..

[7] Gershenwald J.E., Scolyer R.A., Hess K.R., Sondak V.K., Long G.V., Ross M.I., Lazar A.J., Faries M.B., Kirkwood J.M., McArthur G.A. (2017). Melanoma Staging: Evidence-Based Changes in the American Joint Committee on Cancer Eighth Edition Cancer Staging Manual. CA. Cancer J. Clin..

[8] Balch C.M., Gershenwald J.E. (2014). Clinical Value of the Sentinel-Node Biopsy in Primary Cutaneous Melanoma. N. Engl. J. Med..

[9] Huang H., Fu Z., Ji J., Huang J., Long X. (2022). Predictive Values of Pathological and Clinical Risk Factors for Positivity of Sentinel Lymph Node Biopsy in Thin Melanoma: A Systematic Review and Meta-Analysis. Front. Oncol..

[10] Coit D.G., Thompson J.A., Albertini M.R., Barker C., Carson W.E., Contreras C., Daniels G.A., DiMaio D., Fields R.C., Fleming M.D. (2019). Cutaneous Melanoma, Version 2.2019, NCCN Clinical Practice Guidelines in Oncology. J. Natl. Compr. Cancer Netw. JNCCN.

[11] Wong S.L., Faries M.B., Kennedy E.B., Agarwala S.S., Akhurst T.J., Ariyan C., Balch C.M., Berman B.S., Cochran A., Delman K.A. (2018). Sentinel Lymph Node Biopsy and Management of Regional Lymph Nodes in Melanoma: American Society of Clinical Oncology and Society of Surgical Oncology Clinical Practice Guideline Update. J. Clin. Oncol. Off. J. Am. Soc. Clin. Oncol..

[12] Conic R.R.Z., Ko J., Damiani G., Funchain P., Knackstedt T., Vij A., Vidimos A., Gastman B.R. (2019). Predictors of Sentinel Lymph Node Positivity in Thin Melanoma Using the National Cancer Database. J. Am. Acad. Dermatol..

[13] Yonick D.V., Ballo R.M., Kahn E., Dahiya M., Yao K., Godellas C., Shoup M., Aranha G.V. (2011). Predictors of Positive Sentinel Lymph Node in Thin Melanoma. Am. J. Surg..

[14] Mitteldorf C., Bertsch H.P., Jung K., Thoms K.-M., Schön M.P., Tronnier M., Kretschmer L. (2014). Sentinel Node Biopsy Improves Prognostic Stratification in Patients with Thin (pT1) Melanomas and an Additional Risk Factor. Ann. Surg. Oncol..

[15] Murali R., Haydu L.E., Quinn M.J., Saw R.P.M., Shannon K., Spillane A.J., Stretch J.R., Thompson J.F., Scolyer R.A. (2012). Sentinel Lymph Node Biopsy in Patients with Thin Primary Cutaneous Melanoma. Ann. Surg..

[16] Paek S.C., Griffith K.A., Johnson T.M., Sondak V.K., Wong S.L., Chang A.E., Cimmino V.M., Lowe L., Bradford C.R., Rees R.S. (2007). The Impact of Factors beyond Breslow Depth on Predicting Sentinel Lymph Node Positivity in Melanoma. Cancer.

[17] White R.L., Ayers G.D., Stell V.H., Ding S., Gershenwald J.E., Salo J.C., Pockaj B.A., Essner R., Faries M., Charney K.J. (2011). Factors Predictive of the Status of Sentinel Lymph Nodes in Melanoma Patients from a Large Multicenter Database. Ann. Surg. Oncol..

[18] Sassen S., Shaw H.M., Colman M.H., Scolyer R.A., Thompson J.F. (2008). The Complex Relationships between Sentinel Node Positivity, Patient Age, and Primary Tumor Desmoplasia: Analysis of 2303 Melanoma Patients Treated at a Single Center. Ann. Surg. Oncol..

[19] Gershenwald J.E., Thompson W., Mansfield P.F., Lee J.E., Colome M.I., Tseng C.H., Lee J.J., Balch C.M., Reintgen D.S., Ross M.I. (1999). Multi-Institutional Melanoma Lymphatic Mapping Experience: The Prognostic Value of Sentinel Lymph Node Status in 612 Stage I or II Melanoma Patients. J. Clin. Oncol. Off. J. Am. Soc. Clin. Oncol..

[20] Leiter U., Stadler R., Mauch C., Hohenberger W., Brockmeyer N., Berking C., Sunderkötter C., Kaatz M., Schulte K.-W., Lehmann P. (2016). Complete Lymph Node Dissection versus No Dissection in Patients with Sentinel Lymph Node Biopsy Positive Melanoma (DeCOG-SLT): A Multicentre, Randomised, Phase 3 Trial. Lancet Oncol..

[21] Faries M.B., Thompson J.F., Cochran A.J., Andtbacka R.H., Mozzillo N., Zager J.S., Jahkola T., Bowles T.L., Testori A., Beitsch P.D. (2017). Completion Dissection or Observation for Sentinel-Node Metastasis in Melanoma. N. Engl. J. Med..

[22] Keung E.Z., Gershenwald J.E. (2018). The Eighth Edition American Joint Committee on Cancer (AJCC) Melanoma Staging System: Implications for Melanoma Treatment and Care. Expert Rev. Anticancer. Ther..

[23] Santos F.D.M.D., da Silva F.C., Pedron J., Furian R.D., Fortes C., Bonamigo R.R. (2019). Association between Tumor-Infiltrating Lymphocytes and Sentinel Lymph Node Positivity in Thin Melanoma. An. Bras. Dermatol..

[24] Rodriguez Otero J.C., Dagatti M.S., Fernandez Bussy R., Bergero A., Gorosito M., Staffieri R., Villavicencio R., Batalles S.M., Pezzotto S.M. (2019). Sentinel Lymph Node Biopsy in Patients with Thick Primary Cutaneous Melanoma. World J. Oncol..

[25] Swetter S.M., Thompson J.A., Albertini M.R., Barker C.A., Baumgartner J., Boland G., Chmielowski B., DiMaio D., Durham A., Fields R.C. (2021). NCCN Guidelines^®^ Insights: Melanoma: Cutaneous, Version 2.2021. J. Natl. Compr. Cancer Netw..

[26] Ribero S., Osella-Abate S., Sanlorenzo M., Balagna E., Senetta R., Fierro M.T., Macripò G., Macrì L., Sapino A., Quaglino P. (2015). Sentinel Lymph Node Biopsy in Thick-Melanoma Patients (N = 350): What Is Its Prognostic Role?. Ann. Surg. Oncol..

[27] White I., Fortino J., Curti B., Vetto J. (2014). Clinical Impact of Sentinel Lymph Node Biopsy in Patients with Thick (>4 Mm) Melanomas. Am. J. Surg..

[28] Jokic S., Markovic I., Bukumiric Z., Jokic V., Rakovic M., Tripkovic J., Stojiljkovic D., Spurnic I., Jevric M., Matic M. (2018). Predictors of Sentinel Lymph Node Status of Cutaneous Melanoma in Serbian Patients. J. BUON Off. J. Balk. Union Oncol..

[29] Dunne J.A., Wormald J.C.R., Steele J., Woods E., Odili J., Powell B.W.E.M. (2017). Is Sentinel Lymph Node Biopsy Warranted for Desmoplastic Melanoma? A Systematic Review. J. Plast. Reconstr. Aesthetic Surg..

[30] Cheraghlou S., Ugwu N., Girardi M. (2022). Sentinel Lymph Node Biopsy Positivity in Patients with Acral Lentiginous and Other Subtypes of Cutaneous Melanoma. JAMA Dermatol..

[31] Kato J., Hida T., Kamiya T., Horimoto K., Sato S., Sawada M., Minowa T., Handa T., Komatsu S., Uhara H. (2023). Relationships between Tumor Thickness and the Risk of Sentinel Node Metastasis in Acral and Non-Acral Melanoma. Int. J. Dermatol..

[32] Balch C.M., Thompson J.F., Gershenwald J.E., Soong S.-J., Ding S., McMasters K.M., Coit D.G., Eggermont A.M.M., Gimotty P.A., Johnson T.M. (2014). Age as a Predictor of Sentinel Node Metastasis among Patients with Localized Melanoma: An Inverse Correlation of Melanoma Mortality and Incidence of Sentinel Node Metastasis among Young and Old Patients. Ann. Surg. Oncol..

[33] Balch C.M., Soong S., Gershenwald J.E., Thompson J.F., Coit D.G., Atkins M.B., Ding S., Cochran A.J., Eggermont A.M.M., Flaherty K.T. (2013). Age as a Prognostic Factor in Patients with Localized Melanoma and Regional Metastases. Ann. Surg. Oncol..

[34] Featherston C., Nardi W.S., Tomé F.R., Quildrian S.D. (2021). Role of Sentinel Lymph Node Biopsy for Cutaneous Melanoma in Elderly Patients: Preliminary Results in a Latin-American Population. Ecancermedicalscience.

[35] Moody J.A., Ali R.F., Carbone A.C., Singh S., Hardwicke J.T. (2017). Complications of Sentinel Lymph Node Biopsy for Melanoma-A Systematic Review of the Literature. Eur. J. Surg. Oncol. J. Eur. Soc. Surg. Oncol. Br. Assoc. Surg. Oncol..

